# Response inhibition in Parkinson’s disease: a meta-analysis of dopaminergic medication and disease duration effects

**DOI:** 10.1038/s41531-017-0024-2

**Published:** 2017-07-07

**Authors:** Peter Manza, Matthew Amandola, Vivekanand Tatineni, Chiang-shan R. Li, Hoi-Chung Leung

**Affiliations:** 1 Department of Psychology, Stony Brook University, Stony Brook, NY 11790 USA; 2 Department of Psychiatry, Yale University, New Haven, CT 06519 USA; 3 Department of Neuroscience, Yale University, New Haven, CT 06520 USA; 4 Interdepartmental Neuroscience Program, Yale University, New Haven, CT 06520 USA; 5 Beijing Huilongguan Hospital, Beijing, China

## Abstract

Parkinson’s disease is a neurodegenerative disorder involving the basal ganglia that results in a host of motor and cognitive deficits. Dopamine-replacement therapy ameliorates some of the hallmark motor symptoms of Parkinson’s disease, but whether these medications improve deficits in response inhibition, a critical executive function for behavioral control, has been questioned. Several studies of Parkinson’s disease patients “on” and “off” (12-h withdrawal) dopaminergic medications suggested that dopamine-replacement therapy did not provide significant response inhibition benefits. However, these studies tended to include patients with moderate-to-advanced Parkinson’s disease, when the efficacy of dopaminergic drugs is reduced compared to early-stage Parkinson’s disease. In contrast, a few recent studies in early-stage Parkinson’s disease report that dopaminergic drugs do improve response inhibition deficits. Based on these findings, we hypothesized that Parkinson’s disease duration interacts with medication status to produce changes in cognitive function. To investigate this issue, we conducted a meta-analysis of studies comparing patients with Parkinson’s disease and healthy controls on tests of response inhibition (50 comparisons from 42 studies). The findings supported the hypothesis; medication benefited response inhibition in patients with shorter disease duration, whereas “off” medication, moderate deficits were present that were relatively unaffected by disease duration. These findings support the role of dopamine in response inhibition and suggest the need to consider disease duration in research of the efficacy of dopamine-replacement therapy on cognitive function in Parkinson’s disease.

## Introduction

Response inhibition, the ability to suppress a pre-potent or habitual behavioral response, is a critical executive function. Of the various cognitive deficits that patients with Parkinson’s disease (PD) experience (e.g., working memory, planning, and visuospatial attention), response inhibition is of particular importance for the recognition of its negative impact on quality of life.^1–4^ Loss of response inhibition is associated with motor symptom severity^5^ and freezing of gait, a particularly debilitating feature of the disease.^6–9^ With a close link to broader clinical deficits and prediction of later dementia,^10^ response inhibition performance has been posited as a sensitive outcome measure for diagnosis and progression of PD.^11^ Thus, great emphasis has been placed on understanding the neurochemical basis of this deficit, and in particular, the role of dopaminergic medication in response inhibition.

Earlier investigations probed the role of dopaminergic medications, including levodopa, during response inhibition paradigms. However, while seemingly improving other cognitive functions, including task switching and working memory,^12–14^ dopaminergic medications did not appear to provide significant response inhibition benefits to individuals with PD.^15–18^ Some studies in particular suggested that dopamine (DA) deficiency does not underlie poor response inhibition, but postulated a role of other neurotransmitters, such as noradrenaline and serotonin.^19, 20^ Several studies in humans and animals have provided some evidence suggesting that response inhibition is supported by neurotransmitters other than DA.^21, 22^ For instance, atomoxetine, a noradrenaline uptake inhibitor, provides benefit for response inhibition in healthy adults,^23, 24^ adults with attention-deficit hyperactivity disorder (ADHD),^25^ and individuals with PD.^26–28^ In PD, atomoxetine may exert its effects by increasing connectivity of prefrontal circuits critical for response inhibition.^29^ The serotonin-boosting drug citalopram has also shown some beneficial effects on response inhibition in PD.^27, 30^ Studies in rats have come to similar conclusions; while DA transporter inhibition had minor effects on Stop-signal task (SST) performance, atomoxetine improved the stop-signal reaction time (SSRT).^22^ In addition, a recent optogenetics study showed that non-DA cell bodies in the basal forebrain of rats were critical for implementing stopping behavior on the SST.^21^ Together, these studies question a unique role of DA in response inhibition.

However, studies of other specific populations and healthy adults suggests that DA deserves reconsideration in response inhibition. Several studies found that a single dose of methylphenidate (which boosts DA as well as noradrenaline) improved response inhibition behavior in individuals with ADHD,^31^ cocaine dependence^32^ and in healthy adults^33, 34^ (but see^35, 36^). More convincingly, PET studies have found that higher levels of striatal D_1_ and D_2_/D_3_ receptor availability predict better performance on the SST^37, 38^ and that response inhibition performance evokes DA release in prefrontal, parietal, and temporal cortex in healthy adults.^39^

How then, can we reconcile these divergent findings? An important starting place is to consider the characteristics of the PD populations that did not show significant response inhibition improvement with dopaminergic medication.^15–18^ Many previous studies typically include patients in the moderate-to-advanced stages of PD (i.e., individuals with Hoehn and Yahr rating >2 or studies with a wide range of disease duration). This is worth noting because of the profound DA loss in the later stages of PD, and the diminished efficacy of dopaminergic drugs when there are few remaining dopaminergic cells for the drugs to operate on.^40^ Indeed, recent studies provided evidence that early-stage patients with response inhibition impairment seem to benefit from DA treatment.^41, 42^ Thus, studies of patients with moderate-to-advanced PD may not be the best model for examining the role of dopaminergic medication effects on response inhibition.

To re-examine the effects of dopaminergic medications on response inhibition in PD with disease duration in consideration, we performed a meta-analysis of studies in PD populations that used several common response inhibition paradigms (the SST, Go-NoGo, Simon, Flanker, Stroop, and anti-saccade tasks) and looked for an interaction between disease duration and medication status on response inhibition performance while controlling for age effects. We expected to find that studies enrolling patients in the earlier stages (e.g., Hoehn & Yahr I and II or within several years of diagnosis) of PD would show the largest benefit of dopaminergic medication, and hence show performance that was less impaired relative to healthy controls.

## Materials and methods

We performed a literature search using PubMed (www.ncbi.nlm.nih.gov/pubmed) to identify studies that compared a PD cohort with an age-matched healthy adult group on one of six common response inhibition tasks (Stop-Signal, Go-NoGo, Simon, Flanker, Stroop, and Antisaccade tasks). A systematic search up to September 2016 was conducted using the terms [(“Stop-Signal Task” OR “Stop Signal Task” OR “Go-NoGo” OR “Go NoGo” OR “GoNoGo” OR “Go/NoGo” OR “Go No-Go” OR “Antisaccade” OR “Anti-saccade” OR “Simon Task” OR “Flanker” OR “Stroop”) AND (Parkinson OR Parkinson’s)]. This initial search covered a range of studies dated from 1988 to 2016), and only included articles from peer-reviewed journals and written in English. This initial search yielded 305 results. An additional six studies were identified from previous knowledge and recursive reference searching.

We then searched through the text of the 311 studies to screen for: (1) review papers, case studies, book chapters or other non-original research papers; (2) evidence of dementia or surgical intervention; (3) no adequate age-matched control group; (4) atypical versions of the response inhibition tasks that mixed response inhibition with other behavioral contingencies or obvious additional cognitive demands (e.g., Go-NoGo tasks that had a reward component); (5) no information reported on medication status or disease duration for the PD group. Where possible, we included studies that had subsets of data meeting our inclusion criteria. For instance, some studies with a surgical intervention group had an additional PD cohort without surgery; if the non-surgical cohort met our inclusion criteria, we included data from only the non-surgical cohort.

**Fig. 1:**
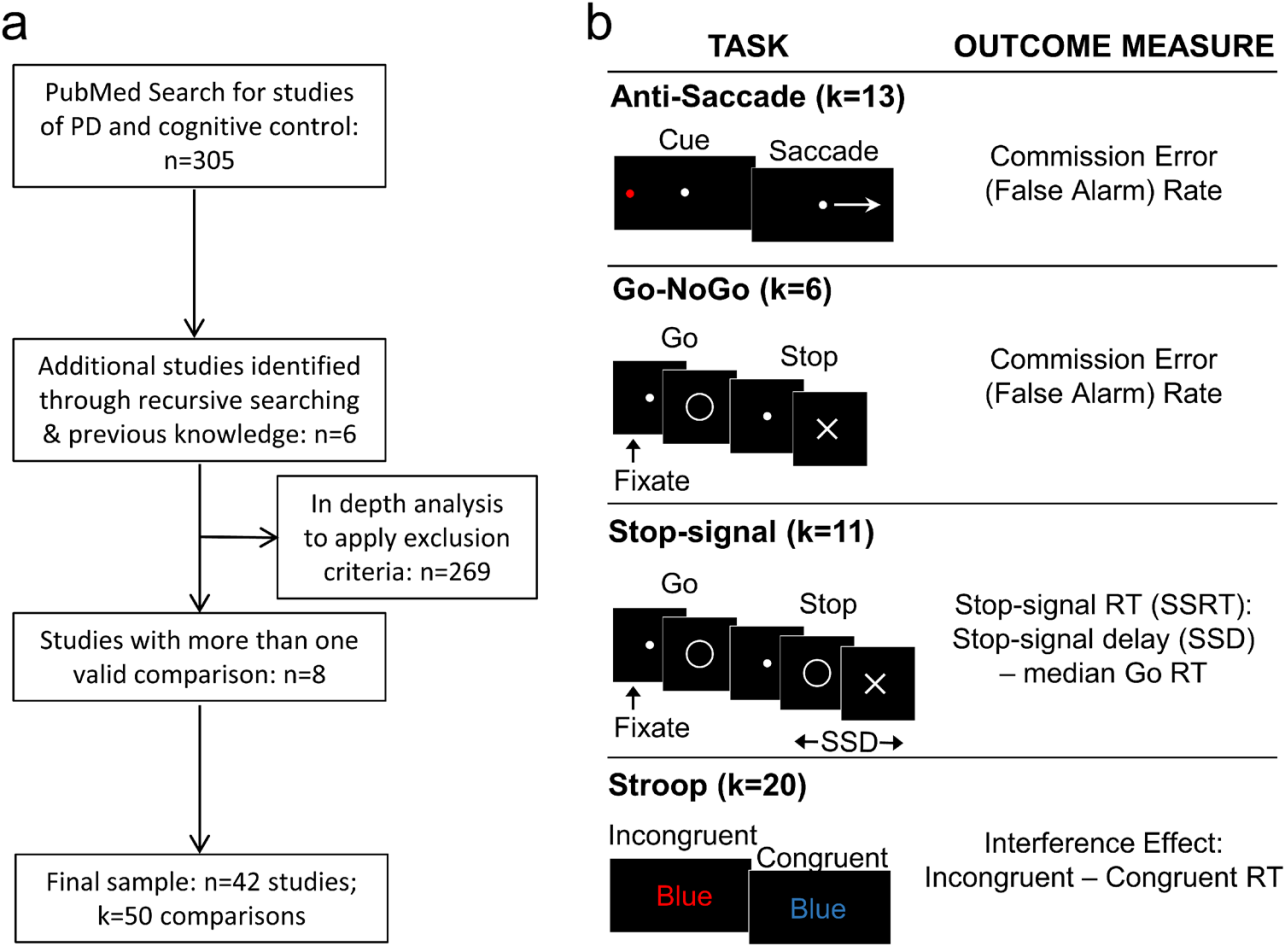
**a** Flow chart of meta-analysis procedure. **b** *Left*: simplified schematic for each task used in the meta-analysis. *Right*: outcome measure used for each task; we chose the measure that was most commonly reported across studies for each task

Forty-seven comparisons from 40 studies remained following application of the exclusion criteria. Corresponding authors were contacted for studies that satisfied all other criteria but did not report sufficient statistics for response inhibition performance, and/or medication status and disease duration. This resulted in the addition of three more comparisons and two more studies. Thus, for the final analysis, data were extracted from 50 comparisons from 42 studies and entered into a spreadsheet (for the full flow-chart, see Fig. 1a). No studies of the Flanker Task or the Simon Task remained after screening for exclusion criteria; thus, we only report on studies of the Anti-Saccade (number of comparisons *k* = 13), Go-NoGo (*k* = 6), SST (*k* = 11), and Stroop tasks (*k* = 20). For analysis, we chose the primary outcome measure of each response inhibition task that was most often reported across studies: for the Anti-Saccade/Go-NoGo tasks, this was the commission error (false alarm) rate, for the SST, the SSRT, and for the Stroop task, the interference effect on response time (i.e., incongruent response time (RT) minus congruent RT; Fig. 1b). We coded studies based on whether patients were taking their dopaminergic medications (“on” medication) or if they underwent a medication washout of at least 12 h (“off” medication) prior to task performance. We also included the average disease duration (years since diagnosis) and mean age of the PD group in data analyses. We chose years since diagnosis as a measure of disease severity instead of Hoehn and Yahr staging for two reasons: first, Hoehn and Yahr staging is not a linear metric and therefore is not suitable for the linear regression analysis we employed (see section on meta-regression in methods below), and second, Hoehn and Yahr staging was not reported for 15 of the studies (and 17 of the 50 comparisons) used here.

We used Comprehensive Meta-Analysis software to perform the meta-analysis.^43^ As in previous studies in this field^44^ we used a random-effects model. We calculated the effect size (Hedges’ g) of mean between-group (PD vs. control) differences in response inhibition performance for each comparison, which corrects for within-study variance that, among other causes, can be a consequence of small sample size. We also assessed the heterogeneity (Q) and inconsistency (I^2^) across studies. Heterogeneity is the ratio of between-study to within-study variance, and inconsistency refers to the ratio of heterogeneity to total variance across studies, i.e. the percentage of heterogeneity that results from differences between studies.^45^ We used the PD vs. control comparisons instead of within-subject comparisons (i.e., direct comparison of patients “on” vs. “off” medications) because too few studies meeting our inclusion criteria reported data from an “on” vs. “off” manipulation (ten studies; see Supplementary Material for a preliminary analysis on these datasets).^43^

Further, we performed meta-regression^46^ to assess the relationship between disease duration and response inhibition performance in the “on” and “off” medication state. Meta-regression carries the same assumptions of a standard regression analysis, except that in the meta-regression here, each data point referred to the effect size of performance difference between the PD group and control group for an entire study, rather than, e.g., across-subject performance on a response inhibition task. Because effect sizes in this analysis were weighted by within-study variation, studies with the highest variability contributed the least to the regression model. In the final analysis, we performed two regressions: one that included the studies with patients “on” medication, and another regression with “off” medication studies. Both regressed average disease duration (years) on the effect size of response inhibition deficit (Hedges’ g). To assess the interaction between disease duration and medication status, we conducted a *z*-test to compare the slopes of the “on” and “off” regressions.^47^ We also conducted additional multiple regression analyses that were identical except that we included the average age of the PD group in each study as a covariate, to account for any potential effects of aging on response inhibition performance that may be independent of medication status and disease duration.^48, 49^ Finally, to account for a possible diminution effect (the finding that effect sizes tend to be larger in older studies and diminish with replication in more recent studies^50^) we performed additional multiple regression analyses while including publication year as a covariate. It would be useful to account for differences in drug dosage amounts across studies by including average levodopa dose equivalency values^51^ as a covariate in the multiple regression analysis; however, many studies do not report these values, and so we were unable to perform formal analysis on all 50 comparisons. Therefore, we performed an analysis on the subset of 14 studies reporting Levodopa-dose (LEDD, see Table 1 for a summary).

**Table 1:** PD vs. healthy control comparisons included in the meta-analysis

Med	Task	Author	Year	N, PD	N, CTRL	Age PD	Age CTRL	% Female PD	% Female CTRL	Duration (Years)	LEDD	MMSE	UPDRS off	UPDRS on	Med status
Off	A-S	Amador^73^	2006	14	11	60.0	55	42.8	66.7	12	N/A	N/A	85	N/A	N/A
Off	A-S	Briand^74^	1999	8	8	73.9	72.6	25	0	8.5	N/A	26.8	29.8	N/A	2 LD, 2 LD+Ag, 1 LD+MAOB, 1 Ag+Chol+MAOB
Off	A-S	Hood^75^ ^a,b^	2007	14	14	59.9	57.79	35.7	42.9	14.7	N/A	27.9	56	31	1 LD, 13 LD+Ag
Off	A-S	Lemos^76^	2016	19	22	67.0	68	36.8	77.3	4	691	29	19	N/A	9 LD, 8 LD+Ag, 1 LD+Chol, 1 LD+Ag+Chol
Off	A-S	Nemanich^77^ ^a^	2016	13	12	68.1	72.3	38.5	66.7	4.7	691	N/A	36.3	N/A	N/A
Off	GNG	Cohen^78^	2014	15	16	67.1	66.6	14.3	62.5	6.5	714	N/A	29.9	N/A	N/A
Off	GNG	Farid^79^	2009	9	9	61.0	59	22.2	11.1	10	1060	N/A	37	11	N/A
Off	GNG	Nemanich^77^ ^a^	2016	13	12	68.1	72.3	38.5	66.7	4.7	691	N/A	36.3	N/A	N/A
Off	SST	George^17^ ^a,b^	2013	16	16	62.6	63.5	50	56.3	4.9	N/A	28.9	41.5	33.68	1 LD, 5 LD+MAOB, 6 LD+MAOB+Ag, 1 LD+Ag+MAOB+COMT, 3 MAOB+Ag
Off	SST	Manza^a,b^	2017	14	37	63.2	64	35.7	40.5	2.8	454	N/A	20.1	14.5	5 LD, 9 LD+MAOB
Off	SST	Obeso^16^ ^a,b^	2011	17	16	69.4	65.69	29	56.3	9.5	915	29.4	30.71	16.25	14 LD, 3 LD+Ag
Off	Stroop	Bohnen^80^	2006	13	14	70.8	N/A	0	N/A	5.9	N/A	28.1	N/A	N/A	N/A
Off	Stroop	Costa^41^ ^a,b^	2014	13	15	68.1	65.5	55	45	2.2	N/A	27.6	16.6	N/A	9 LD, 9 LD+Ag/MAOB, 2 Ag
Off	Stroop	Fera^81^ ^a,b^	2007	12	10	59.9	55.2	50	50	3.5	489	28.4	18	11.5	4 LD, 8 LD+Ag
On	A-S	Bonnet^82^	2014	21	27	54.8	36.2	38.1	8	9.6	N/A	27.6	25.9	N/A	20 LD/Ag
On	A-S	Cameron^83^	2010	14	12	60.1	59.5	28.6	58.3	4.9	N/A	29.1	24.1	N/A	1 LD, 5 LD+Ag, 4 Ag, 4 LD+other
On	A-S	Harsay^84^	2010	20	18	61.8	69.4	0.5	50	7	N/A	N/A	N/A	16.7	9 LD, 6 MAOB, 3 COMT, 14 Ag
On	A-S	Hood^75^ ^a,b^	2007	14	14	59.9	57.79	35.7	42.9	14.7	N/A	27.9	56	31	1 LD, 13 LD+Ag
On	A-S	Rivaud-Péchoux^85^	2007	15	10	64.3	64	26.7	N/A	6.6	N/A	N/A	N/A	15.2	7 LD, 5 LD+Ag, 3 LD+Chol
On	A-S	Van Koningsbruggen^86^	2009	19	20	66.6	66.1	31.6	N/A	7.1	N/A	>27	N/A	14.1	5 LD, 6 LD+Ag, 5 Ag, 1 Ag+MAOB, 1Ag+COMT
On	A-S	Van Stockum^87^	2008	18	18	65.7	66.3	33.3	33.3	8.8	N/A	N/A	N/A	N/A	1 LD, 7 LD+Ag, 4 LD+MAOB, 5 LD+Ag+MAOB, 1 Other
On	A-S	Walton^7^	2015	34	38	66.4	63.9	32	50	6.5	N/A	28.2	22.45	N/A	N/A
On	GNG	O’Callaghan^88^	2013	25	15	64.5	64.2	36	20	7.3	897	28	12.9	N/A	6 LD, 14 LD+Ag, 2 LD+COMT, 1 Ag, 2 Ag+MAOB
On	GNG	Tachibana^62^	1997	29	19	63.9	59.7	70	N/A	6	N/A	27.2	N/A	N/A	4 LD, 2 LD+Ag, 5 LD+Ag+other, 10 LD+other, 2 other, 6 none
On	GNG	Ye^27^ ^a^	2014	21	20	64.0	65.3	47.6	40	10.8	633	28.9	N/A	20.6	LD+Ag+MAOB
On	SST	Claassen^56^	2015	12	12	60.8	58.5	N/A	50	6.1	520	29	N/A	15.7	LD+Ag
On	SST	Gauggel^2^	2004	32	32	57.8	56.7	46.9	45	9	N/A	N/A	N/A	N/A	LD+Ag+Chol
On	SST	George^17^ ^a,b^	2013	16	16	62.6	63.5	50	56	4.9	N/A	28.9	41.5	33.7	1 LD, 5 LD+MAOB, 6 LD+MAOB+Ag, 1 LD+Ag+MAOB+COMT, 3 MAOB+Ag
On	SST	Manza^a,b^	2017	14	37	63.2	64	35.7	40.5	2.8	454	N/A	20.1	14.5	5 LD, 9 LD+MAOB
On	SST	Obeso^16^ ^a,b^	2011	17	16	69.4	65.7	29	56	9.5	915	29.4	30.7	16.3	14 LD, 3 LD+Ag
On	SST	Rae^28^	2016	19	10	69.4	67.4	31.5	50	9.8	1080	28.5	N/A	25.9	LD
On	SST	Stefanova^89^	2014	36	33	64.7	65	N/A	7.6	10.1	720	27.9	N/A	32.3	LD+Ag
On	SST	Ye^27^ ^a^	2014	21	20	64.0	65.3	47.6	40	10.8	633	28.9	N/A	20.6	LD+Ag
On	Stroop	A’Campo^90^	2012	35	29	65.5	64.2	43	46.3	6	N/A	27.4	N/A	N/A	N/A
On	Stroop	Bohlhalter^91^	2009	12	12	59.1	N/A	33.3	N/A	7.7	766	28.5	N/A	N/A	N/A
On	Stroop	Brown^92^	1988	16	16	60.3	58.9	31.3	N/A	11.6	566	N/A	N/A	N/A	10 LD, 2 LD+C hol, 4 LD+other
On	Stroop	Costa^41^ ^a,b^	2014	13	15	68.1	65.5	55	55	2.2	N/A	27.6	16.6	N/A	9 LD, 9 LD+Ag/MAOB, 2 Ag
On	Stroop	Fera^81^ ^a,b^	2007	12	10	59.9	55.2	50	50	3.5	489	28.4	18	11.5	4 LD, 8 LD+Ag
On	Stroop	Hanes^93^	1996	26	25	62.6	44	44	46	7	N/A	N/A	N/A	N/A	20 LD, 5 Other
On	Stroop	Hsieh^94^	2008	26	27	63.5	N/A	37	N/A	3.3	N/A	N/A	N/A	N/A	N/A
On	Stroop	Kierzynka^95^	2011	37	40	61.0	63.2	59.5	35	6.2	N/A	28.7	N/A	28.9	LD+Ag+Chol+MAOB
On	Stroop	McNamara^96^	2003	24	15	70.2	67.3	0	0	8	N/A	27	N/A	N/A	N/A
On	Stroop	Müller-Oehring^97^	2015	11	11	63.0	62	54.5	64	3.1	473	N/A	N/A	9.5	6 LD+Ag+MAOB, 1 Ag+MAOB, 3 MAOB, 1 none
On	Stroop	Ranchet^98^	2011	25	25	65.4	66.7	24	N/A	6.4	N/A	28.1	N/A	14.9	LD + Ag + COMT + MAOB
On	Stroop	Ranchet^99^	2013	19	21	66.1	69.1	10.5	19	7.5	742	N/A	N/A	16.4	LD + Ag + MAOB
On	Stroop	Relja^100^	2006	25	25	63.0	62.5	40	44	5.4	N/A	28.2	N/A	N/A	N/A
On	Stroop	Vandenbossche^9^	2012	14	14	68.0	66.3	21	21	8.2	N/A	28.4	N/A	33.2	N/A
On	Stroop	Wild^101^	2013	18	18	69.3	69.4	55.6	44	8.4	N/A	26.4	N/A	16.2	LD + Ag
On	Stroop	Witt^102^	2006	22	22	58.0	56.9	27.3	41	8.1	599	N/A	N/A	16.6	N/A
On	Stroop	Woodward^103^	2002	30	34	69.3	69.9	53.3	65	5.8	N/A	>23	N/A	N/A	N/A

	N, PD	N, CTRL	Age PD	Age CTRL	% Female PD	% Female CTRL	Duration (Years)	LEDD	MMSE	UPDRS OFF	UPDRS ON
Average OFF	14.0	15.1	65.6	64.4	33.1	49.4	6.7	714	28.3		
Average ON	21.2	20.7	63.8	62.3	36.9	40.6	7.2	678	28.2		
Average A-S	17.2	17.2	63.7	62.2	31.2	45.1	8.4	691	28.1	39.3	21.6
Average GNG	18.7	15.2	64.8	64.5	38.1	40.1	7.6	799	28.0	29.0	15.8
Average SST	20.0	22.3	64.2	63.6	37.6	45.3	7.3	712	28.9	36.1	24.3
Average Stroop	20.2	19.9	64.6	62.5	37.2	41.7	6.0	589	27.9	17.3	17.6

Fifty comparisons were used from 42 studies

*Med* Medication state (On or Off dopaminergic medications), *CTRL* healthy controls, *LEDD* Levodopa-dose equivalency, *MMSE* Mini-mental state examination, *A-S* Antisaccade, *GNG* Go-NoGo, *SST* Stop-Signal Task, *LD* Levodopa, *Chol* Anticholinergic, *Ag* DA Agonist, *MAOB* MAO-B inhibitor, *COMT* COMT inhibitor, *N/A* not available

^a^ more than one contrast was included from the same study

^b^ denotes that the study has a within-subject design “on” vs. “off” medication; for “Med Status”, each value refers to how many individuals were taking that combination of medications (no numbers are listed if there was insufficient information to derive individual medication profiles in the original report)

### Data availability

The datasets analysed during the current study were acquired from the references listed in Table 1 and are available from the corresponding author on reasonable request.

## Results

The final sample included 50 comparisons from 42 studies, which are described in Table 1.

**Fig. 2:**
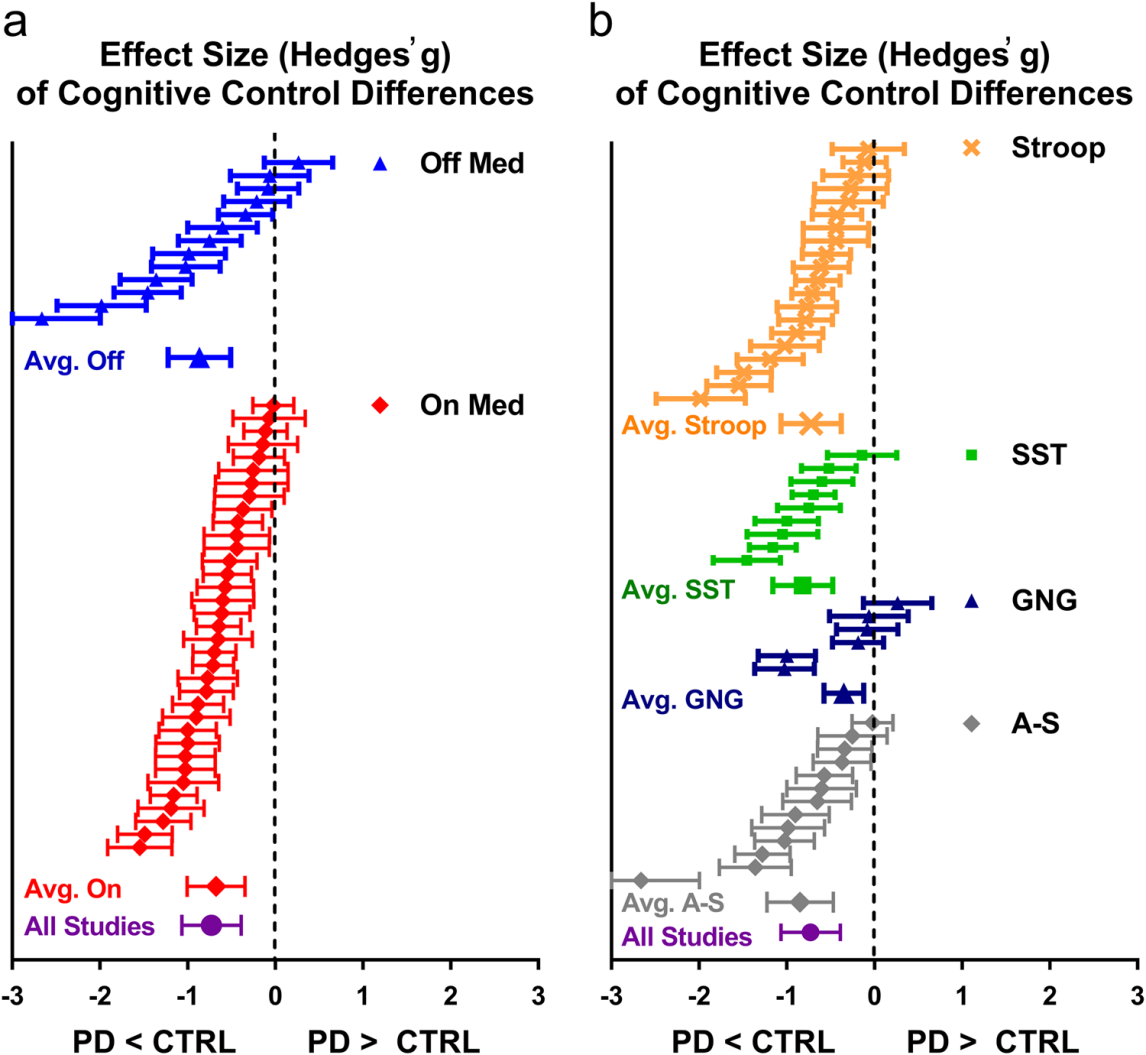
Forest plot of effect sizes for all studies that compared performance on a response inhibition task between a PD group and a matched healthy control group. **a** Studies sorted by medication status. **b** Studies sorted by task. Effect sizes to the *left* of the *vertical dashed line* indicate that performance of the PD group was poorer than controls. Note: Avg., Average; SST, Stop-Signal Task; GNG, Go-NoGo; A-S, Anti-Saccade

To assess differences in response inhibition performance for PD on and off medication in comparison to healthy control, we extracted means and standard deviations from the PD and control groups from the critical outcome measure of each task: SSRT for the SST, false alarm rate for Go-NoGo and Anti-saccade tasks, and the Stoop interference effect (i.e., incongruent RT minus congruent RT); all outcome measures were continuous variables. For five studies of which standard deviations were not reported, we extracted the mean for each group, or the mean difference between groups, and the *t*-value or *p*-value of the PD vs. control comparison to calculate effect sizes. Table 1 describes the samples included from each study, including average age and disease duration of the PD group. Effect sizes for each comparison are shown in the Forest plot in Fig. 2. The average effect size for “on” and “off” samples was moderate and significant, suggesting that patient groups with PD showed response inhibition deficits relative to their matched controls (“off” Hedges’ *g* = −.86; “on” Hedges’ *g* = −.66; all studies; Hedges’ *g* = −.72, all *p*’s < 10^−6^). There was no significant difference in effect size between “on” and “off” samples, two-sample *t*-test: *t*(48)* = *.88, *p = *.38, and post-hoc *t*-tests indicated no significant “on” vs. “off” differences within any of the four tasks (all *p’s > *.05). The average effect size for the Anti-saccade (Hedges’ *g* = −.85), SST (Hedges’ *g* = −.75), and Stroop (Hedges’ *g* = −.72) tasks were moderate and significant (*p*’s < 10^−6^), while the average Go-NoGo effect size was not (Hedges’ *g* = −.35; *p = *.09). However, there was no significant difference in effect size across the four tasks; One-way analysis of variance: *F(*3,46) = 1.50; *p* = .23.

While the vast majority of studies reported poorer performance among the PD groups relative to each of their control group (48 out of 50 comparisons), effect sizes were variable across studies. We found 35 out of 50 studies that showed a significant effect size for PD vs control group differences in response inhibition performance. There was significant heterogeneity in the “off” and “on” samples (“off” sample: *Q* = 37.24, *p < *.001, *I* ^*2*^ = 65.09; “on” sample: *Q* = 57.07, *p = *.01, *I* ^*2*^ = 38.68), but heterogeneity did not significantly differ by medication status (*Q* = .47, *p = *.49). There was significant heterogeneity in the Anti-saccade (*Q* = 28.44, *p < *.01, *I* ^*2*^ = 57.80), Go-NoGo (*Q* = 11.61, *p < *.05, *I* ^*2*^ = 56.94) and Stroop tasks (*Q* = 34.90, *p < *.02, *I* ^*2*^ = 45.53), but not the SST (*Q* = 15.58, *p = *.11, *I* ^*2*^ = 35.83). However, heterogeneity did not significantly differ across the four tasks (*Q* = 4.26; *p* = .24).

**Fig. 3:**
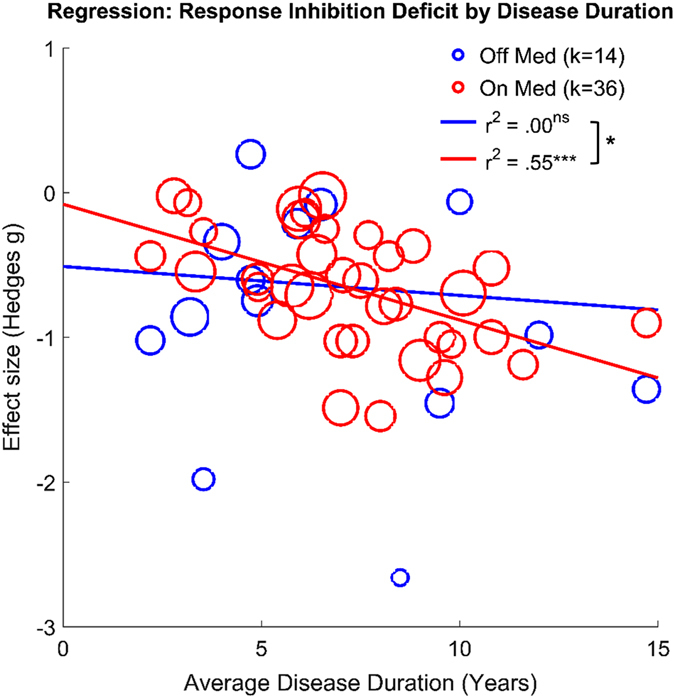
Regression plot of response inhibition deficits on average disease duration for the “off” medication (*blue*) and “on” medication (*red*) samples. Effect sizes less than 0 indicate that PD patients demonstrated poorer response inhibition performance than healthy controls. Each *bubble* represents a comparison from one study, weighted by within-study variance. *Smaller bubbles* represent studies with higher variance than others and thus, have less influence on the regression. The regression line of best fit for each sample is also shown. The difference in slopes was significant (*z* test; *p = *.04), indicating that “off” medication, deficits were moderate and relatively unaffected by disease duration, whereas “on” medication, deficits were significantly associated by disease duration. *p < .05; ***p = .001

Next, we performed meta-regression to examine how the effect size of PD vs. control group differences in response inhibition varied across studies with relation to disease duration for each medication status. For “on” vs. control comparisons, the effect of disease duration was significant (*z* = −3.24, *r* ^2^ = .55, *p* = .001), while the effect was not significant for “off” vs. control comparisons (*z* = −0.84, *r* ^2^ = .00, *p* = .40; Fig. 3). In other words, in the “on” medication state, patients with longer average disease duration showed more severe response inhibition deficits, whereas the deficits were relatively unaffected by disease duration when patients were “off” dopaminergic medication. Disease duration thus explained more variance in response inhibition deficits across studies with participants “on” medication compared to the “off” medication studies, with the test for differences in slopes significant (*z = *−2.05, *p = *.04). The same pattern of disease duration effects remained even when age was controlled for in the regression analysis. Specifically, effect of age was not significant for both on and off medication state (*z*’s < 0.8, *p*’s > .42), with disease duration remaining significant in the “on” sample (*z* = −3.12, *r* ^2^ = .47, *p* = .002) but again not significant in the “off” sample (*z* = −.79, *r* ^2^ = .00, *p* = .43); the slope difference between “on” and “off” samples remained significant (*z* = −2.05, *p* = .04). To explore the potential role of medication dosage in the meta-regression results described above, we examined the association of LEDD for the 14 studies in the “on” state that included an average LEDD score. Such analysis showed negligible effects of LEDD when included in a model together with disease duration (effect of disease duration: *z* = −2.35, *p* = .018; effect of LEDD: *z* = 0.38, *p = *.705), or when simply including LEDD as the only predictor variable (*z* = 0.39, *p* = .70, *R* ^2^ = 0.00). There was a significant effect of publication date for both the “on” and “off” samples, such that earlier studies showed larger effect sizes between PD and controls (i.e., the diminution effect; *z*’s > 2.3, *p*’s < .05). However, including publication year as a covariate did not change the primary findings: the effect of disease duration remained significant in the “on” sample (*z* = −3.20, *p* = .001) and not significant in the “off” sample (*z* = .67, *p* = .51); and slope difference between “on” and “off” samples remained significant (*z* = −5.55, *p* < .001).

## Discussion

The primary results from the meta-analysis demonstrated that individuals with PD show deficits in response inhibition performance, specifically on tasks that test cognitive control of behavioral responses, including SST, Go-NoGo, Anti-saccade, and Stroop tasks. These deficits were of moderate effect size and significant for both the “on” and “off” medication samples, comparable to those in a previous meta-analysis examining a broader range of cognitive tasks in PD.^44^ However, our meta-analysis revealed a differential effect of dopaminergic medication status by disease duration on response inhibition performance across studies. Specifically, “off” medication, patient groups tended to show moderate deficits relative to controls regardless of disease duration, but “on” medication, disease duration predicted the severity of response inhibition deficit. Thus, in the earlier stages of PD, patients on dopaminergic medication tend to show performance that is more comparable to healthy control levels.

These results suggest that disease duration is an important factor when considering medication effects on response inhibition performance in PD. Previous work has compared dopaminergic effects on response inhibition directly within subjects “on” and “off” medication and emphasized null findings,^15–18^ but these studies tended to include patients with moderate to advanced PD (e.g., individuals with Hoehn & Yahr rating >2 or studies with a wide range of disease duration). We suggest that these studies observed no significant effects of DA-replacement therapy because of severe loss of dopaminergic midbrain cells in the later stages of PD.^52, 53^ It is routinely observed that dopaminergic drugs lose their efficacy in more advanced PD,^40^ putatively because diminishing numbers of DA cells render few targets for the drugs to exert their effects. Although the evidence is indirect, the current findings provide a starting point to suggest that the role of DA in response inhibition deserves reconsideration, particularly in early-stage PD. Thus, a critical next step is to directly test dopaminergic medication effects on response inhibition in a within-study design with patients in the early stages of PD.

It is important to consider the possibility that impulse control disorders may play a role in these findings. That is, advanced PD patients “on” medication show poor response inhibition due to increased likelihood of having impulse control disorders. It is estimated that 15–20% of individuals using dopaminergic medications long-term, especially DA agonists, are susceptible to developing impulse control problems over time.^54^ However, several lines of evidence suggest that impulse control problems would not be the most likely contributor to response inhibition deficits in PD. Recent studies have delineated separable neural and behavioral substrates of motivational vs. cognitive/motor impulsivity (e.g., gambling addiction vs. stop-signal inhibition^55^). While PD patients with impulse control disorders are prone to compulsive motivational behaviors including gambling, shopping, sex, and binge-eating,^54^ they do not demonstrate increased cognitive/motor impulsivity on the Stroop and SST compared to matched PD patients without impulse control disorder.^56, 57^ Further, acute withdrawal of dopaminergic medication did not help improve impulsive error rates relative to the “on” medication state in the Simon task.^42, 58^ Finally, although DA agonists specifically have been implicated in impulse control disorders in PD, a recent investigation by van Wouwe and colleagues^42^ found no significant differences on Simon task performance between PD groups on agonist monotherapy vs. levodopa monotherapy or combination therapy. Thus, the type of impulsivity described with long-term medication use in PD does not map on well to the type of impulsivity investigated in the response inhibition literature. Still, longitudinal studies are needed to determine more definitively whether long-term medication use impairs response inhibition in PD.

Our findings correspond with several recent studies that found an association between dopaminergic function and response inhibition performance in both healthy adults and clinical populations.^31–34, 37–39^ There are some notable exceptions, however: some studies in healthy adult humans^59, 60^ and rats^22, 61^ have found that pharmacologically manipulating DA transmission had minor effects on response inhibition performance. There are several possible explanations for these seemingly discrepant findings. For one, it is possible that there are subtle differences in how DA supports performance on different response inhibition tasks. Indeed, average response inhibition deficits in PD were fairly similar for Antisaccade, SST, and Stroop, but not Go-NoGo (average Hedges’ g: Antisaccade: −.85; Go-NoGo: −.35; SST: −.75; Stroop task: −.72), perhaps due to differences in task difficulty. For instance, no significant group differences were observed when accuracy was >95% for both groups in one Go-NoGo study,^62^ whereas SSRT deficits can be profound on challenging SSTs that induce roughly 50% error rates on stop trials^2, 15^; this is in accord with recent suggestions that Go-NoGo may not be sufficiently sensitive to detect response inhibition deficits in PD.^11^ While a previous meta-analysis demonstrated highly overlapping neural correlates of Go-NoGo and SST, differences were also noted;^63^ and others have also noted that response inhibition performance does not always correlate neatly between tasks,^64^ perhaps because of differences in task demand and/or performance strategy.^11^ Notably, effect sizes for the antisaccade task were large in our meta-analysis, despite the fact that this was the only task not requiring hand movements. Thus, response inhibition deficits in PD are not simply due to motor deficits in the most-affected extremities. Our findings that SST effect sizes were rather large and, compared to the other three tasks, had the least heterogeneity across studies, suggests that the SST may be a more sensitive task for identifying response inhibition deficits and drug effects. Another possible reasons for discrepant findings on DA’s role in response inhibition may be related to baseline levels of DA function. Previous attempts to boost DA transmission in healthy adults^59, 60^ might not improve response inhibition performance because most healthy adults should presumably already have optimal levels of DA to support task performance, based on the inverted U-shape theory of dopaminergic function in cognition.^65^ In support, Colzato et al.^66^ found that L-Tyrosine (DA precursor) administration improved SSRT only in the subset of healthy adults with the T/T polymorphism of the dopamine D2 receptor, which confers low levels of striatal DA. These considerations highlight the need for systematic assessment of DA’s role in response inhibition in various populations, using a variety of behavioral tasks and medication statuses.

While this meta-analysis suggests an association between dopaminergic medication and response inhibition performance, there is substantial evidence that other neurotransmitters also play a critical role in these behaviors in PD. Animal studies have found that drugs which alter noradrenaline and serotonin can have marked effects on response inhibition behavior.^67^ This has prompted researchers to find non-dopaminergic therapies for response inhibition deficits in PD. Recent studies have reported some success with noradrenergic and serotonergic drugs in SST performance in moderate-to-advanced PD.^26, 27, 68^ A current challenge is thus to find optimal combination therapies that might promote response inhibition in PD. Given the current findings on how disease duration interacts with DA-replacement therapy, it seems likely that medication regimes for cognition may need to be adjusted throughout the course of the disease. These results also highlight the therapeutic potential to restore the deteriorating dopaminergic system in PD^69^; our study suggests this might benefit response inhibition in addition to the primary motor symptoms of PD.

There are a number of limitations to acknowledge. While all meta-analyses must deal with many sources of heterogeneity across independent studies, studies of PD may involve additional sources of variance that cannot all be accounted for. The current study examined the role of disease duration and medication status in cognitive task performance, and controlled for the possible effects of age and publication date. Yet, many variables are not consistently reported could explain additional variance, such as drug type, dosage differences, motor subtype and global assessments of cognitive function. Future reports should strive to include levodopa dose equivalency values,^51^ as well as comprehensive data on motor (Unified Parkinson’s Disease Rating Scale^70^) and cognitive (e.g., Montreal Cognitive Assessment^71^) function to help account for these variables. Other clinical differences across patient groups may also obscure findings, such as levels of comorbid depression and fatigue, which are common in PD and may relate to response inhibition performance. Studies with “on” vs. “off” medication within-subject designs would be important, as they at least control for subject variability within studies. To date, there were insufficient numbers of these studies that met our inclusion criteria (*k* = 10) to perform the critical meta-analysis using within-subject comparisons (preliminary analyses on these data are reported in Supplementary Material, which are generally in line with the conclusions of the primary analysis presented here). In addition, it should be noted that “off” medication groups are typically defined by a 12-h washout procedure. While this is sufficient to produce significant differences in motor symptom severity from the medicated state, significant loss of motor function can continue for weeks after discontinuation of medication in early-stage PD.^72^ To our knowledge no studies have examined changes in response inhibition performance follow a similar timecourse with medication washout. Future work is needed to determine what the optimal washout duration is for probing dopaminergic function in response inhibition. Another limitation regards sample size. This is because we chose to select only studies that used relatively pure cognitive versions of response inhibition tasks, and hence could not report on the many studies that used variations on these paradigms, e.g. reward-based Go-NoGo tasks. Nonetheless, these findings supplement previous analyses^44^ that explored how dopaminergic medications are related to cognitive deficits in PD more broadly.

Overall, results from this meta-analysis suggest in PD, response inhibition deficits are least severe relative to controls when patients are in early-stages of PD and “on” dopaminergic medications. Deficits are more severe in later stages of PD “on” medications, and under medication withdrawal, regardless of disease duration. This pattern of findings provides indirect evidence that dopaminergic medications may support response inhibition in early-stage PD.

## Electronic supplementary material

Supplementary Material
