# Supplementary material for: Response inhibition in Parkinson’s disease: a meta-analysis of dopaminergic medication and disease duration effects
Source: NPJ Parkinsons Dis. 2017 Jul 7;3:23. doi: 10.1038/s41531-017-0024-2 (PMC5501877; doi:10.1038/s41531-017-0024-2)
Supplement: Supplementary file 1 — Supplementary Material [file 41531_2017_24_MOESM1_ESM.docx]

**Supplementary Results**. To more directly examine how disease duration may interact with medication status in PD, we examined data from the 10 studies that met our inclusion criteria and reported data comparing individuals with PD “off” vs “on” medication using a within-subject design,. This sample was inadequately powered for a reliable meta-regression analysis and had an uneven distribution of disease duration. Nevertheless, we conducted a preliminary meta-regression and it yielded an effect (Supplementary Figure 1) in the same direction as shown in Figure 3 of the main text. In studies with shorter average disease duration, patients tended to show better response inhibition performance in the “on” relative to the “off” medication state, whereas in studies with longer average disease duration, there was less of a benefit from medication (*z* = 1.54, *p* = .12; *r*^2^ = .35). Across all 10 studies, there was a significant benefit of medication on response inhibition performance (random effects model; *z* = 4.21, *p* < .001). This was driven by the studies with shorter average disease duration. That is, using median split, the 5 studies with “short” average disease duration of 2.97 years had significantly greater medication-based benefits in response inhibition performance than the 5 studies with “long” average disease duration of 9.05 years; Hedges’ g = -.64 “short” vs. -.21 “long” (two-sample *t­*-test, *p* = .030). See Supplementary Table 1 for more details about these 10 studies.

**Supplementary Figure 1**. Regression plot of response inhibition deficits on average disease duration for the ten studies that included a direct “off” vs. “on” medication comparison using a within-subject design (see **Supplementary Table 1** for a summary). Effect sizes less than 0 indicate that PD patients demonstrated poorer response inhibition performance when “off” compared to “on” medication. Each bubble represents a comparison from one study, weighted by within-study variance. Smaller bubbles represent studies with higher variance than others and thus, have less influence on the regression. The regression line of best fit is also shown. Note that all studies, especially those with low average disease duration, have a negative effect size, a potential effect predicted from the meta-regression analysis of “on” versus control groups.


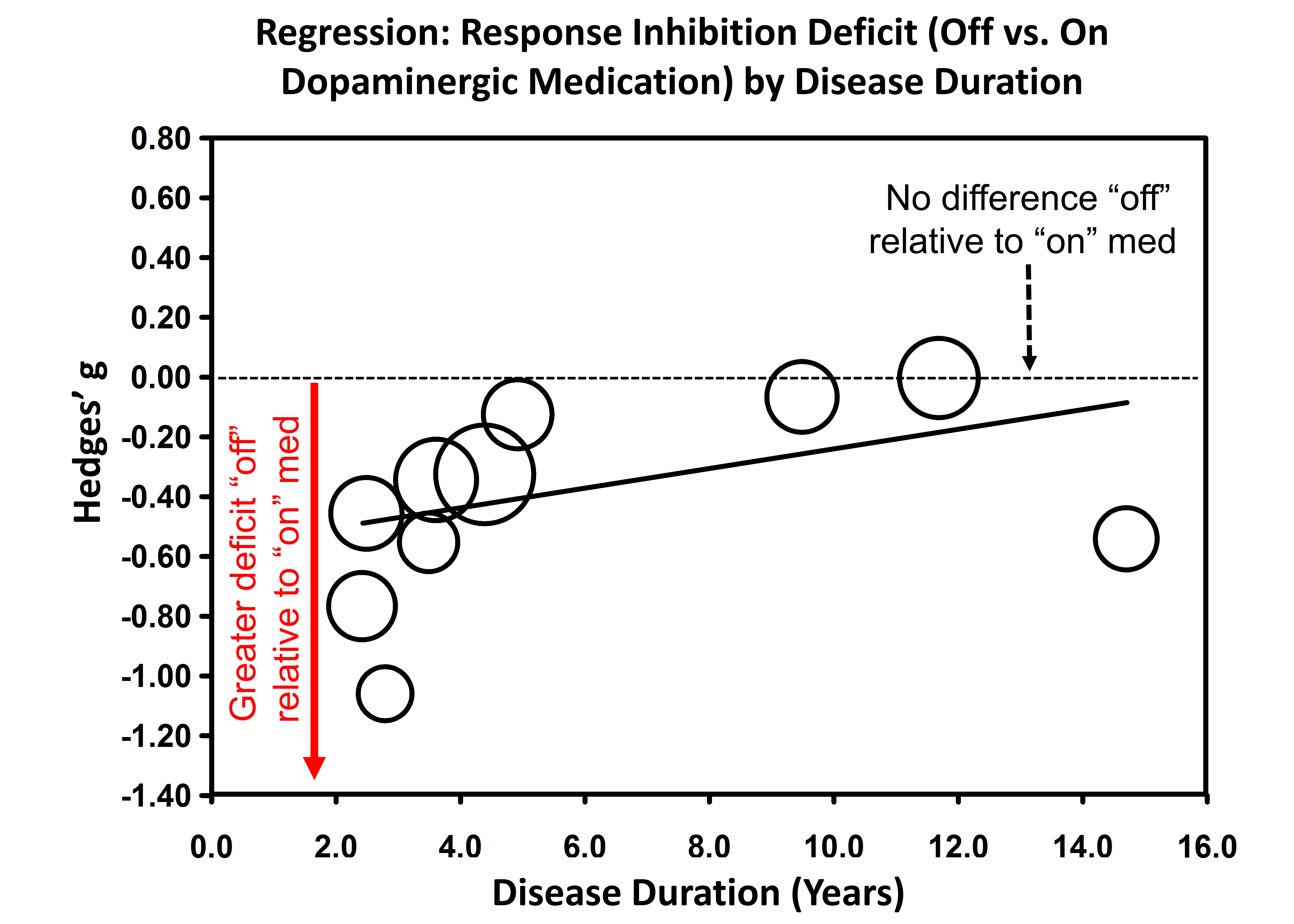


**Supplementary Table 1**. Studies containing a within-subject “on” vs. “off” design that fit inclusion criteria and report all key statistics for differences in response inhibition performance.

| **First Author Last Name, Date** | **Task** | **Age** | **Disease Duration (Years)** | | **UPDRS On** | **UPDRS Off** | **LEDD** | **MMSE** | **Hedges' g (effect size)** |
| --- | --- | --- | --- | --- | --- | --- | --- | --- | --- |
| Cameron, 2012 | Antisaccade^1^ | 64.2 | | 3.62 | 23.2 | 28.6 | 517.39 | 29 | -0.35 |
| Hood, 2007 | Antisaccade^2^ | 59.9 | | 14.71 | 31 | 56 | N/A | 27.89 | -0.54 |
| Van Wouwe, 2016 | Simon^3^ | 63.7 | | 4.4 | N/A | 26.9 | 732 | N/A | -0.33 |
| George, 2013 | SST^4^ | 63.5 | | 4.93 | 33.68 | 41.5 | N/A | 28.94 | -0.13 |
| Manza, Under Review | SST^5^ | 63.2 | | 2.8 | 14.5 | 20.14 | 454 | N/A | -1.06 |
| Obeso, 2011 | SST^6^ | 69.4 | | 9.5 | 16.25 | 30.71 | 915.94 | 28.11 | -0.07 |
| Brusa, 2003 | Stroop^7^ | 57.0 | | 2.5 | 19.45 | 29.34 | N/A | N/A | -0.46 |
| Costa, 2014 | Stroop^8^ | 68.8 | | 2.43 | N/A | 16.6 | 336 | 27.6 | -0.77 |
| Fera, 2007 | Stroop^9^ | 59.9 | | 3.5 | 11.5 | 18 | 488.6 | 28.4 | -0.56 |
| Djamshidian, 2011 | Stroop^10^ | 64.2 | | 11.7 | 14.4 | 26.8 | 821 | N/A | -0.01 |

Supplementary References

1. Cameron, I. G. M. *et al.* Impaired executive function signals in motor brain regions in Parkinson’s disease. *NeuroImage* **60,** 1156–70 (2012).

2. Hood, A. J. *et al.* Levodopa slows prosaccades and improves antisaccades: an eye movement study in Parkinson’s disease. *Journal of Neurology, Neurosurgery & Psychiatry* **78,** 565–570 (2007).

3. van Wouwe, N. C. *et al.* Dissociable Effects of Dopamine on the Initial Capture and the Reactive Inhibition of Impulsive Actions in Parkinson’s Disease. *Journal of Cognitive Neuroscience* **28,** 710–723 (2016).

4. George, J. S. *et al.* Dopaminergic therapy in Parkinson’s disease decreases cortical beta band coherence in the resting state and increases cortical beta band power during executive control. *NeuroImage: Clinical* **3,** 261–270 (2013).

5. Manza, P. *et al.* Levodopa restores response inhibition and neurobehavioral signatures of proactive control in Parkinson’s disease. *Under Review*

6. Obeso, I., Wilkinson, L. & Jahanshahi, M. Levodopa medication does not influence motor inhibition or conflict resolution in a conditional stop-signal task in Parkinson’s disease. *Experimental Brain Research* **213,** 435–445 (2011).

7. Brusa, L. *et al.* Pramipexole in comparison to 1-dopa: A neuropsychological study. *Journal of Neural Transmission* **110,** 373–380 (2003).

8. Costa, A. *et al.* Dopamine treatment and cognitive functioning in individuals with Parkinson’s disease: the ‘cognitive flexibility’ hypothesis seems to work. *Behavioural neurology* **2014,** 260896 (2014).

9. Fera, F. *et al.* Dopaminergic modulation of cognitive interference after pharmacological washout in Parkinson’s disease. *Brain Research Bulletin* **74,** 75–83 (2007).

10. Djamshidian, A., O’Sullivan, S. S., Lees, A. & Averbeck, B. B. Stroop test performance in impulsive and non impulsive patients with Parkinson’s disease. *Parkinsonism & Related Disorders* **17,** 212–214 (2011).
